# Navigating Trade‐Offs in Early Math: How Informational Priming Influences Parent–Child Interaction

**DOI:** 10.1111/cdev.70031

**Published:** 2025-08-21

**Authors:** Linxi Lu, Marina Vasilyeva, Elida V. Laski

**Affiliations:** ^1^ University of Chicago, Harris School of Public Policy and Committee on Education Chicago Illinois USA; ^2^ Boston College, Lynch School of Education and Human Development Chestnut Hill Massachusetts USA

**Keywords:** home math environment, informational priming, parent‐child interaction

## Abstract

Home math interventions often incorporate informational priming—explicit prompts emphasizing parental math input. While effective in increasing math talk, its impact on child outcome is mixed. This study examined how informational priming shapes the content and dynamic of math interactions. In year 2023, 122 Chinese parent–child dyads ($M_{\text{ChildAge}\_\text{Years}}$ = 5.25, 52% girls) participated in a business‐as‐usual play session, followed 2 weeks later by another session after parents were briefed on math talk importance. Parents increased math talk quantity and diversity but provided less autonomy support and exerted more control (large effects). Meanwhile, children were more frequently disengaged (medium effect). The findings reveal trade‐offs, highlighting the need to balance increased math input with motivational support to foster children's math development.

Parent–child interactions play a significant role in shaping children's early math development (Elliott and Bachman 2018) that, in turn, serve as a key predictor of later academic achievement and career success (Duncan et al. 2007). Even before the start of formal education, substantial variability exists in children's math skills (DeFlorio and Beliakoff 2015; Duncan and Magnuson 2011; Larson et al. 2015), highlighting the need to identify factors that support early math learning. In response, researchers have developed interventions aimed at increasing parental math input to foster children's math development (e.g., Doss et al. 2022; Douglas et al. 2024; Leyva et al. 2023; Vandermaas‐Peeler et al. 2012). Many of these interventions employ informational priming—explicit prompts emphasizing the importance of math talk—as a mechanism to enhance parental math input (e.g., Vandermaas‐Peeler et al. 2012).

While such interventions have successfully increased the quantity and diversity of parental math talk, their impact on children's math outcomes remains inconsistent. Some studies report positive effects (e.g., Dulay et al. 2019); whereas others find no significant improvements (e.g., Leyva et al. 2023) or even unintended negative consequences, such as reduced child engagement or lower math performance (e.g., Mayer et al. 2023; Tian et al. 2025). These mixed findings suggest a need to comprehensively examine not only how informational priming influences parental math input but also how it shapes broader parent–child interactional dynamics. Using a within‐subject experimental design, the present study investigates whether increasing parents' awareness of the importance of math input enhances the quantity and diversity of parental math input, while also exploring potential trade‐offs between the amount of math talk and the nature of the support provided.

## 1 Parental Math Talk and Early Math Development

The home math environment (HME) plays a pivotal role in shaping children's math development, with parental math talk serving as one of its central components (Daucourt et al. 2021). Parental math talk can be broadly categorized into two domains: numerical and spatial. Numerical talk—conversations about numbers, quantities, and mathematical operations—has been extensively studied and shown to predict children's numeric understanding both concurrently and longitudinally (Casey et al. 2018; Ramani et al. 2015; Son and Hur 2020; Susperreguy and Davis‐Kean 2016). In contrast, spatial talk—conversations about shapes, sizes, distances, and spatial relations—has received less attention but is increasingly recognized for its role in shaping children's spatial reasoning and broader mathematical achievement (Ferrara et al. 2011; Levine et al. 2012; Lombardi and Dearing 2021; Ribeiro et al. 2020). While observational studies examining both numerical and spatial aspects of parental math talk within a single sample are scarce, a few studies relying on parent self‐report suggest that caregivers tend to engage more frequently in numeracy activities than in spatial ones (Zippert and Rittle‐Johnson 2020). This pattern may stem, in part, from parents' conceptualization of math as primarily number‐focused—a tendency identified in previous work (Lu et al. 2025a). A similar pattern has been observed in early childhood classrooms, where math instruction has been found to largely focus on numeracy as opposed to spatial concepts (Bachman et al. 2018; Scalise et al. 2025).

Both numerical and spatial math talk show substantial variability across parents, which has been linked to families' socioeconomic status (SES). Studies conducted in both the U.S. (cf. Dearing et al. 2022) and China (e.g., Lu et al. 2025a) consistently demonstrate that, during play interactions, higher‐SES parents tend to engage preschoolers in math talk with greater frequency and diversity. This SES difference may stem, in part, from low‐SES parents having greater math anxiety (Berkowitz et al. 2021; Elliott and Bachman 2018). It may also reflect differences in parents' tendency to spontaneously notice and talk about math‐relevant aspects of the environment associated with educational level (Lu et al. 2025b).

The variability in parental math talk, both across and within SES groups, and its relation to the development of early math knowledge, have motivated researchers to design interventions aimed at enhancing the quantity and quality of math‐related input children receive at home. The following section examines these interventions, discussing different approaches to increasing parental math input and their implications for children's learning outcomes.

## 2 Informational Priming to Increase Parental Math Input

Existing interventions to enhance parental math input often employ structured materials, such as math‐themed picture books (e.g., Eason and Ramani 2020; Gibson et al. 2020; Purpura et al. 2021; Wang et al. 2024), digital applications (e.g., Berkowitz et al. 2015; Mayer et al. 2023), and math‐related board games (e.g., Cheung and McBride 2017; Tian et al. 2025). Other interventions encourage parents to integrate math talk into specific daily activities, such as cooking (e.g., Vandermaas‐Peeler et al. 2012), grocery shopping (e.g., Hanner et al. 2019), or museum visits (e.g., Braham et al. 2018). A third category takes a broader approach, prompting parents to incorporate more math talk into their everyday interactions (e.g., Doss et al. 2022; Douglas et al. 2024; Leyva et al. 2023; Mayer et al. 2023).

A common feature across many of these interventions is the use of informational priming—explicit messaging or prompts that emphasize the importance of early math learning and parent–child math interactions—to promote greater math talk. For example, some studies have used signs highlighting the link between parental talk and children's academic success (e.g., Hanner et al. 2019), while others have provided parents with direct instructions to focus on math prior to joint activities (e.g., Eason and Ramani 2020; Vandermaas‐Peeler et al. 2012). Additionally, text message reminders have been employed to encourage parents to engage in math‐related discussions with their children (e.g., Douglas et al. 2024; Leyva et al. 2023; Mayer et al. 2023). These strategies have proven effective in increasing the quantity and diversity of parental math talk, with parents in experimental conditions producing more math‐related talk than those in the control conditions (e.g., Eason and Ramani 2020; Vandermaas‐Peeler et al. 2012).

Despite the well‐established successes in increasing parental math talk, the impact of such interventions on children's math development has been inconsistent. While some studies report positive effects on children's math knowledge (e.g., Dulay et al. 2019), others find no significant differences between intervention and control groups (e.g., Leyva et al. 2023; Doss et al. 2022). Alarmingly, some interventions have even yielded unintended negative effects. For instance, Mayer et al. (2023) found that text messages emphasizing the importance of parental math talk had a negative impact on children's math performance postintervention. Similarly, an intervention combining parental information sessions with joint spatial activities resulted in lower engagement and poorer spatial vocabulary performance among children in the intervention group compared to controls (Tian et al. 2025). These mixed findings raise important questions about whether such interventions may inadvertently alter parent–child interaction dynamics in ways that undermine their intended benefits.

Moreover, most informational priming interventions designed to enhance the home math environment have been conducted in Western contexts, but their effects may vary across cultural settings with differing parenting values and practices. In China, for instance, early academic achievement is strongly emphasized, and parenting tends to be more directive (Pan et al. 2006), which may influence how parents interpret and respond to such interventions. Despite a thorough literature search, we identified only one study that examined an intervention resembling informational priming in the Chinese context (Gao 2024). In this study, parents of young children (0–3 years old) received text messages highlighting developmental milestones and encouraging parent–child interactions. Consistent with some prior findings from the U.S. (e.g., Leyva et al. 2023; Doss et al. 2022), the intervention led to increased parental time investment in joint activities but did not produce measurable improvements in child outcomes. As this study focused on general developmental topics among very young children, it remains unclear how informational priming may influence Chinese parents' engagement in specific academic domains such as mathematics.

## 3 Potential Trade‐Offs Between Parental Math Talk and Interactional Dynamics

Beyond the quantity and diversity of parental math talk, other aspects of parental input during parent–child interactions—particularly the amount and type of regulatory talk—may play a role in shaping children's engagement and math development. According to Self‐Determination Theory (SDT; Ryan and Deci 2000), children's intrinsic motivation and academic performance are influenced by three fundamental psychological needs: autonomy, competence, and relatedness. Parental autonomy support—encouraging children to take the lead, make choices, and explore activities independently—fosters a sense of competence and sustained engagement. In contrast, controlling behaviors—issuing directives without justification or pressuring children to comply—can undermine motivation and confidence. Empirical research supports these claims, showing that parental autonomy support is positively associated with children's intrinsic motivation for learning math (Aunola et al. 2013; Hagger et al. 2015) and is predictive of both concurrent and long‐term math achievement (Oh et al. 2022; Rispoli et al. 2019; Silinskas and Kikas 2019). Conversely, excessive parental control has been linked to lower math achievement, diminished motivation, and reduced interest in math‐related activities (Oh et al. 2022; Retanal et al. 2021; Su et al. 2015).

While informational priming has shown promise in increasing parental math talk, it may inadvertently alter the nature of parent–child interactions. Research suggests that when tasks are framed as academically relevant, parents may experience heightened anxiety, reduce autonomy support, and become more controlling. For example, Kulkofsky (2011) found that mothers provided less autonomy support when a reminiscing task was framed as an instructional exercise rather than a bonding opportunity. Similarly, Grolnick et al. (2002) demonstrated that mothers became more controlling when informed that their child would be tested after a joint poem‐reading task. Huang et al. (2023) further observed that both Chinese mothers and fathers provided less autonomy support during structured activities, such as worksheets or applied problem‐solving tasks, compared to math‐related games.

This potential trade‐off—where increased parental math talk comes at the cost of reduced autonomy support and heightened control—may help explain why many home‐based interventions successfully enhance parental math input but fail to produce corresponding gains in children's math skills. Given the multidimensional nature of parent–child math interactions, it is critical to examine how informational priming simultaneously affects both parental math input and interactional dynamics. However, to our knowledge, only one study has partially explored these two dimensions in tandem.

Eason and Ramani (2020) asked parents to engage their preschool children in playing with toys provided by researchers. In the guided condition, parents received written instructions on how to incorporate math talk into play, whereas in the unguided condition, parents were asked to play as they normally do. The results showed that parents produced more math talk in the guided condition, compared to unguided condition; at the same time, there was no difference between conditions in parental reports of how engaging they thought the interaction was. The authors acknowledge that the two contexts varied in multiple dimensions (general emphasis on math was supplemented by specific wording prompts and additional materials), making it difficult to determine which of these factors affected parents' math talk and their perception of engagement. Further, since interactional dynamics were measured only with parental self‐report, it remains to be determined whether children would also find both contexts to be equally engaging, and it may be useful to incorporate behavioral measures of engagement.

An additional question is how the informational priming may influence parents from diverse socio‐economic backgrounds. Given documented SES‐related differences in parental math talk, math anxiety, and frequency of early math activities (e.g., Lu et al. 2025a; Vandermaas‐Peeler et al. 2009), lower‐SES families may face challenges in responding to informational priming (e.g., it may active math anxiety or make them feel unprepared to discuss math‐related topics with their children). Thus, including families from different SES backgrounds may provide valuable insights for designing interventions that maximize both cognitive and motivational benefits.

## 4 Present Study

The present study examined how informational priming might impact the content and the dynamics of parent–child math interactions. Using a within‐subject experimental design, parents engaged in two separate play sessions with their children. Both sessions involved preparing for an imaginary party, a scenario known to naturally elicit math talk without being explicitly math‐focused (Lu et al. 2023). The key difference between the two contexts was that in the first (uninformed) context, parents were instructed to interact with their children as they usually would; whereas in the second (informed) context, they received informational priming emphasizing the importance of math talk for children's development. We hypothesized that this informational priming would heighten parents' awareness of the mathematical opportunities within the play activity, leading to observable changes in their math‐related and regulatory talk during the interaction. This hypothesis generated two sets of predictions, as illustrated below.

The first set of predictions focused on the effects of informational priming on parental math talk (quantity and diversity) produced by the two experimental contexts. Building on prior research showing that math‐relevant informational priming promotes parental math talk (e.g., Braham et al. 2018; Eason and Ramani 2020), we predicted that parents in the informed context would engage in more math talk than in the uninformed context (Hypothesis 1). Additionally, we expected the effect of informational priming to vary by math domain. Parents have been found to focus primarily on numerical concepts, overlooking other relevant areas (e.g., spatial reasoning), when defining early math (Lu et al. 2025a). Thus, we expected the effect of informational priming would be more pronounced on numerical talk than on spatial talk (Hypothesis 2).

Our second prediction concerned differences in parental regulatory talk and children's engagement between the two contexts. Based on existing research (e.g., Huang et al. (2023); Kulkofsky 2011), we predicted that the informed context would elicit more parental control and less autonomy support compared to the uninformed context (Hypothesis 3). Furthermore, given the well‐established associations between autonomy support, control, and engagement (e.g., Oh et al. 2022), we predicted that children would be less engaged in the informed context (Hypothesis 4).

In addition to the main predictions, we explored several questions concerning relations between child and family characteristics on parental behavior. Regarding the impact of child age on parent–child interactions, we hypothesized that parents of older children would engage in more math talk, provide greater autonomy support, and exhibit less controlling behavior (Hypothesis 5). This expectation is based on the possibility that parents adapt their behaviors to align with their child's development, which reflects the bi‐directional nature of parent–child interactions (Loulis and Kuczynski 1997). With respect to parental education, we hypothesized that it would be related to parents' math talk based on the evidence that more educated parents tend to produce more math talk compared to less educated parents (c.f., Dearing et al. 2022) (Hypothesis 6). Regarding the relation between parental education and regulatory talk, we did not have a specific hypothesis, given the mixed findings in previous research on the association between SES and autonomy support (c.f., Distefano and Meuwissen 2022).

## 5 Method

### 5.1 Participants

Participants included 124 parent–child dyads recruited from southeast China via parent networking platforms and community organizations during 2023; 2 dyads were not included in the analyses because they did not complete the second session. Post hoc sensitivity analysis using G*Power (Version 3.1; Faul et al. 2007) revealed that this sample size has 80% power for detecting a small to medium effect (*d* = 0.26) at a significance level of *α* = 0.05. Of the 122 dyads whose interactions were analyzed, 98 (80%) of the parents were mothers and 61 (50%) of the children were girls. Children's age ranged from 40 to 84 months (*M* = 62.68, SD = 11.05). The families came from diverse SES backgrounds, with 34.4% reporting less than high school education, 16.4% some high school education, 45.1% a two‐ or four‐year college degree, and 4.1% a graduate degree. Across the sample, the average amount of parental education was 12.30 years, with the range from 3 to 19 years (SD = 3.44). All families had the same ethnicity (Han), language (Mandarin), and living arrangement (both parents lived with the child). All children attended kindergarten at the time of the study. In China, children typically enter kindergarten at the age of three and transition to first grade 3 years later at the age of six.

### 5.2 Design and Procedure

Each parent–child dyad participated in two play sessions that were 2 weeks apart. Both sessions were conducted in the same quiet, convenient location chosen by the family—school conference room (66%), research lab (21%), participants' home (11%), or a neighborhood garden (2%). During both sessions, parents were provided with a set of toys that included 15 unique plastic fruits and vegetables as well as a plastic plate (see Figure S1A, for materials and instructions). Pilot interviews with families from similar populations indicated that this type of play is familiar to and commonly practiced by parent–child dyads. Based on authors' previous work, the game and materials also naturally provide opportunities for math‐related talk without being explicitly math‐focused (Lu et al. 2023).

Parents were asked to play for 10 min their child just as they typically would. The main difference between the play sessions was whether parents received informational priming. In the first session (*uninformed context*), parents were unaware of the study's focus on math; at the beginning of the second session (*informed context*), they were told: “Talking about math during play is really helpful for children's early math development.” Further, to mitigate toy familiarity effects, a different set of 15 fruits and vegetables were used in the second session. In addition to the two play sessions, parents completed a demographic questionnaire (after the first session) that included items on parental education (in years), child age (in months), and parent and child gender (1 = male, 0 = female).

### 5.3 Coding

All interactions were video recorded, transcribed, and coded to assess (a) the quantity and quality of parental math talk (b) parental regulatory talk, and (c) children's engagement throughout the play activities. For parental math and regulatory talk, coding was based on the verbatim transcription of the parent–child interactions, in which the flow of speech was divided into utterances using the algorithm developed by Huttenlocher et al. (2010). To assess child engagement, we relied on the video recordings to consider nonverbal behavior and facial expressions along with verbalizations to allow for more accurate inferences regarding engagement levels. The transcription and coding processes were conducted by independent observers who were not familiar with the study's research questions or hypotheses. Twenty dyads were randomly selected from each session (16% of data in total) to establish interrater reliability. The interclass correlation coefficient values for each aspect of parent–child interactions included in Table S1A,B, indicated good to excellent reliability across raters (Koo and Li 2016). Any cases of disagreements were resolved through discussion with the research team.

### 5.4 Parental Math Talk

Utterances containing numerical and spatially related questions or statements were coded using a scheme that included 16 types of math talk, adapted from Klibanoff et al. (2006). Definitions and examples for each code are provided in Table S1A. The quantity of math talk was defined as the total number of instances in which parents referenced math concepts, while the diversity measure reflected the number of distinct math concepts introduced during the interaction. For each parent, four measures of math talk were computed, reflecting either quantity or diversity of (a) numerical utterances and (b) spatial utterances.

### 5.5 Parental Regulatory Talk

Utterances involving either of the two types of regulatory language—autonomy support and control—were coded using a scheme developed in prior research (Laurin and Joussemet 2017). Detailed descriptions and examples of each type are provided in Table S1B. For each parent, two measures of regulatory talk were computed, reflecting the total number of utterances in which parents provided (a) autonomy support or control.

### 5.6 Child (Dis)Engagement

The video recordings were divided into 30‐s intervals, and each interval was coded for child engagement or disengagement. It should be noted that traditional engagement scales (e.g., Wood et al. 2016) typically classify child behaviors along a continuum from actively engaged to passively engaged to disengaged. However, these frameworks may not fully capture cultural norms in which children are encouraged to passively follow parental guidance (e.g., Park and Lau 2016). For example, a child who sits quietly, listens attentively, and follows parental instructions without questions or other interruptions may be considered passively engaged in a Western context but actively engaged in Chinese traditions. To ensure a more culturally sensitive approach, we coded only active disengagement. A binary measure classified any 30‐s interval as “disengaged” if the child exhibited one or more of the predefined behaviors, such as walking away, looking away, or refusing to respond. The child's disengagement score was calculated as the proportion of intervals in which disengagement occurred.

### 5.7 Data Analysis Approach

The data were analyzed using IBM SPSS Statistics (Version 28.0). Preliminary analyses were conducted to generate descriptive statistics for all key measures; Pearson's and Spearman's correlational analyses were used to examine within‐ and between‐context correlations among the primary variables. Further, as a manipulation check to assess whether the informational priming specifically influenced math and regulatory talk—rather than more generally affecting the overall amount of parental speech—we compared the total amount of parental talk across conditions. Finally, to test our primary hypotheses, we used repeated‐measures ANCOVAs (with context—uninformed versus informed—as within subject factor). The models included child age and gender, as well as parental education and gender, as covariates in the ANCOVAs to determine whether parental education and child age might impact parent–child interactions. Significant ANCOVAs were followed up by post hoc simple‐effects tests (with Bonferroni corrections); significant covariates were followed up by linear regressions within each context.

This study was primarily confirmatory in nature; hypotheses regarding the effects of informational priming on parental math talk, regulatory language, and child engagement were prespecified. Additional analyses examining moderators (e.g., parental education, child age) and interactional patterns were exploratory.

## 6 Results

### 6.1 Descriptive and Correlational Analyses

Table 1 presents the means and standard deviations for the key study variables; Table 2a provides correlations among these variables within each context (uninformed and informed). As shown in Table 2b, the measures of math and regulatory talk were positively associated with the overall amount of parental talk in each context. The quantity and diversity measures of numeric talk were also positively associated with each other in both contexts, but the two subtypes of math talk (numeric and spatial) were not significantly correlated in either context. Further, the quantity of numeric talk was positively associated with the use of control. Lastly, in the informed context, the use of control was positively associated with child disengagement.

**TABLE 1:** Means, standard deviations for key study variables (*N* = 122).

Key variables	Uninformed context (M, SD)	Informed context (M, SD)
Overall talk		
Total utterance	169.74 (68.80)	163.26 (58.52)
Math talk		
Numeric talk (Quantity)	5.74 (8.05)	39.78 (25.48)
Numeric talk (Diversity)	1.98 (1.68)	5.80 (3.01)
Spatial talk (Quantity)	6.97 (6.82)	7.04 (9.32)
Spatial talk (Diversity)	1.52 (0.96)	1.11 (0.95)
Regulatory talk		
Autonomy support	12.08 (8.68)	6.08 (7.17)
Control	16.72 (11.16)	22.83 (15.81)
Child disengagement		
% of time	7 (13)	11 (19)

**TABLE 2a:** Correlations for the key variables in uninformed context (*N* = 122).

	Demographic		Overall talk	Math talk				Regulatory talk		Child engagement
	1	2	3	4	5	6	7	8	9	10
**Demographic**										
1. Child Age	—									
2. Parent education	−0.19*	—								
**Overall talk**										
3. Total utterance	−0.19*	0.43***	—							
Math talk										
4. Numeric talk (Quantity)	−0.03	0.18*	0.24**	—						
5. Numeric talk (Diversity)	−0.02	0.26**	0.32***	0.85***	—					
6. Spatial talk (Quantity)	−0.03	0.11	0.36***	0.18*	0.14	—				
7. Spatial talk (Diversity)	0.02	0.13	0.27**	0.10	0.09	0.49***	—			
**Regulatory talk**										
8. Autonomy Support	−0.07	0.27**	0.45***	0.20*	0.24**	0.02	0.11	—		
9. Control	0.00	0.05	0.46***	0.23*	0.26**	0.13	0.15	0.07	—	
**Child disengagement**										
10. % of time	−0.01	0.94	−0.16	0.01	−0.02	−0.18*	−0.16	−0.00	−0.15	—

*Note:* †*p* < 0.10, *p < 0.05, **p < 0.01, ***p < 0.001.

**TABLE 2b:** Correlations for the key variables in informed context (*N* = 122).

	Demographic		Overall talk	Math talk				Regulatory talk		Child engagement
	1	2	3	4	5	6	7	8	9	10
**Demographic**										
1. Child age	—									
2. Parent education	−0.19*	—								
**Overall talk**										
3. Total utterance	−0.10	0.20*	—							
**Math talk**										
4. Numeric talk (Quantity)	0.10	−0.05	0.45***	—						
5. Numeric talk (Diversity)	0.28**	0.03	0.28**	0.74***	—					
6. Spatial talk (Quantity)	0.00	−0.02	0.19*	0.04	0.00	—				
7. Spatial talk (Diversity)	0.06	0.05	0.07	0.14	0.15	0.57***	—			
**Regulatory talk**										
8. Autonomy support	−0.16	0.25***	0.31***	−0.12	−0.03	0.10	0.01	—		
9. Control	−0.18*	−0.09	0.51***	0.30***	0.13	0.11	0.11	0.01	—	

10. % of time	−0.10	−0.08	−0.007	−0.02	−0.05	−0.04	0.01	0.15	0.23*	—

*Note:* †*p* < 0.10, *p < 0.05, **p < 0.01, ***p < 0.001.

Next, we assessed the consistency of parental behaviors across the two contexts by conducting Pearson's and Spearman's correlations for each outcome variable (see Table 3). Significant correlations were observed for the total number of parental utterances, both types of regulatory talk, and child disengagement (all *p's* < 0.001). However, none of the parental math talk measures, except the diversity of spatial talk, showed significant correlations between the two contexts (all *p's* > 0.05).

**TABLE 3:** Correlations of key variables between two contexts (*N* = 122).

Key variables	Pearson's	Spearman's
Total utterance	0.33***	0.49***
Numeric talk (Quantity)	0.10	0.01
Numeric talk (Diversity)	0.17	0.08
Spatial talk (Quantity)	0.07	0.11
Spatial talk (Diversity)	0.25**	0.22*
Autonomy support	0.63***	0.55***
Control	0.39***	0.41***
Child disengagement (% of time)	0.48***	0.32***

*Note:* †*p* < 0.10, *p < 0.05, **p < 0.01, ***p < 0.001.

### 6.2 Inferential Analysis

#### 6.2.1 Overall Amount of Parental Talk

To establish parents' baseline input and determine whether context had a general effect on the amount of parental verbal input, we first examined difference by contexts in the overall amount of parent talk. A repeated‐measure ANCOVA on the total number of parent utterances revealed no differences across contexts, *F*(1, 119) = 0.46, *p* = 0.50, $\eta_{p}^{2}$ = 0.01. Parental education had an effect on the quantity of parental talk, *F*(1, 119) = 18.65, *p* < 0.001, $\eta_{p}^{2}$ = 0.14, with more educated parents producing a greater number of utterances. Further, there was an interaction between context and parental education, *F*(1, 119) = 8.50, *p* = 0.004, $\eta_{p}^{2}$ = 0.07. To investigate the nature of the interaction, we used regression analyses, given that parental education was a continuous variable, to examine the relation between parental education and overall amount of parental talk within each context (see Table S1C, for regression models). In the uninformed context, parental education was a significant predictor of the overall amount of parental talk (*β* = 0.42, *p* < 0.001), with the model accounting for a significant proportion of variance (Adjusted $R^{2}$ = 0.19, *p* < 0.001). In the informed context, while parental education was a significant predictor (β = 0.20, *p* = 0.03), it did not account for a significant proportion of variance (Adjusted $R^{2}$ = 0.03, *p* = 0.14).

#### 6.2.2 Parental Math Talk

##### 6.2.2.1 Quantity

**FIGURE 1:**
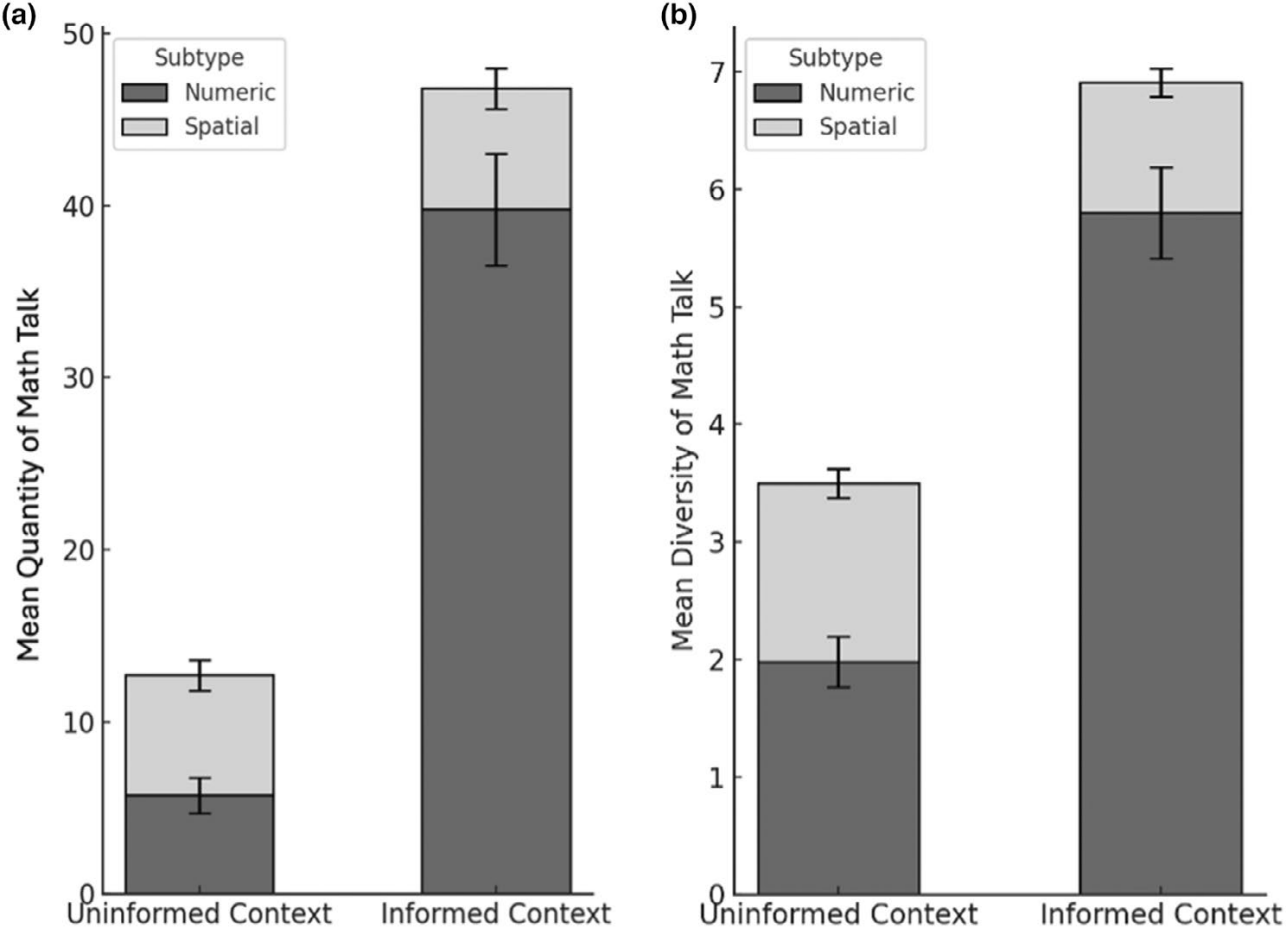
Parental math talk by context and type (M, SE). (a) Quantity of math talk by context and type. (b) Diversity of math talk by context and type.

To test Hypothesis 1 concerning the effects of context on the amount of math talk and Hypothesis 2 concerning the differential effects of contexts on numerical and spatial talk, we conducted a 2 (context: uninformed vs. informed) × 2 (kind of math talk: numeric vs. spatial) repeated‐measures ANCOVA. The results are shown in Figure 1a. The analysis revealed a main effect of context, *F*(1,117) = 188.80, *p* < 0.001, $\eta_{p}^{2}$ = 0.61. Average parent math talk was almost four times greater in the informed play context (*M* = 46.82, SD = 27.48) than in the uninformed play context (*M* = 12.64, SD = 11.40). There was also a main effect of kind of math talk, *F*(1, 117) = 157.04, *p* < 0.001, $\eta_{p}^{2}$ = 0.57. The average number of numeric utterances parents produced (*M* = 45.52, SD = 27.44) was more than four times greater the number of their spatial utterances (*M* = 8.56, SD = 9.45). Moreover, there was an interaction between context and kind of math talk, *F*(1, 117) = 166.34, *p* < 0.001, $\eta_{p}^{2}$ = 0.58. Follow‐up simple‐effects tests with Bonferroni corrections indicated that the amount of numeric talk was significantly greater in the informed, compared to the uninformed context (*p* < 0.001), whereas the amount of spatial talk was comparable in the two contexts (*p* = 0.94). With respect to covariates, there was an effect of parent gender on math talk, *F*(1,117) = 5.53, *p* = 0.02, $\eta_{p}^{2}$ = 0.05: mothers (*M* = 62.84, SD = 31.37) provided more math talk than fathers (*M* = 45.98, SD = 31.45). Given the unequal distribution of mothers (80%) and fathers (20%) in the sample, findings related to parental gender should be interpreted with caution. None of the remaining covariates, nor their interactions with context or kind of math talk, contributed to the variation.

To control for the loquacity of parents, we conducted a parallel ANCOVA, using the proportion of math talk (out of the total number of utterances) as the outcome variable. The analysis produced the same pattern of results: (a) a main effect of context: *F*(1,117) = 232.06, *p* < 0.001, $\eta_{p}^{2}$ = 0.66, whereby the proportion of math utterances was greater in the informed (*M* = 29%) than in the uninformed context (*M* = 8%) (b) a main effect of kind of math talk: *F*(1, 117) = 157.94, *p* < 0.001, $\eta_{p}^{2}$ = 0.57, with the proportion of numerical talk (*M* = 14%) more three times greater than the proportion of spatial talk (*M* = 4%); and (c) an interaction between context and kind of math talk, *F*(1, 117) = 172.75, *p* < 0.001, $\eta_{p}^{2}$ = 0.59. Follow‐up simple‐effects tests with Bonferroni corrections showed that the proportion of numerical talk was greater in the informed, compared to the uninformed context (*M* = 25% vs. 5%, *p* < 0.001), whereas the proportion of spatial talk was comparable in the two contexts (*p* = 0.56). The results support our hypotheses that informational priming increases the quantity of parental math talk, and that its effect is more pronounced for numerical than for spatial talk.

##### 6.2.2.2 Diversity

Parallel analyses were conducted to examine differences in the diversity of parental math talk (Hypothesis 1 and 2). The results are shown in Figure 1b. The 2 (context: uninformed vs. informed) x 2 (kind of math talk: numeric vs. spatial) repeated‐measures ANCOVA revealed a main effect of context, *F*(1,117) = 120.86, *p* < 0.001, $\eta_{p}^{2}$ = 0.50. Parents referenced almost twice as many unique math concepts in the informed than in the uniformed context: *M* = 6.90, SD = 3.29 versus *M* = 3.48, SD = 2.01, respectively. The analysis also revealed a main effect of kind of math talk, *F*(1, 117) = 237.24, *p* < 0.001, $\eta_{p}^{2}$ = 0.66: parents referenced more numeric concepts than spatial ones, *M* = 3.89, SD = 1.88 versus *M* = 1.31, SD = 0.77. Moreover, there was significant interaction between context and kind of math talk, *F*(1, 117) = 189.65, *p* < 0.001, $\eta_{p}^{2}$ = 0.61. Post hoc comparisons indicated that parents used a greater diversity of numeric talk in the informed, compared to uninformed, context (*p* < 0.001, $\eta_{p}^{2}$ = 0.31). The reverse pattern was observed for spatial talk: parents used a less diverse range of spatial talk in the informed, compared to the uninformed context (*p* < 0.001, $\eta_{p}^{2}$ = 0.05).

With respect to covariates, aligned with hypothesis 5 and 6, both parental education, *F*(1,117) = 6.03, *p* = 0.02, $\eta_{p}^{2}$ = 0.05, and child age, *F*(1,117) = 7.66, *p* = 0.007, $\eta_{p\;}^{2}$ = 0.06, contributed to the variance in the diversity of math talk—parents with more years of education and those interacting with older children generated more diverse math talk. Additionally, there was an interaction between context and child age, *F*(1,117) = 7.28, *p* = 0.008, $\eta_{p}^{2}$ = 0.06. Post hoc regression analyses (see Table S1D) showed that child age predicted the diversity of math talk in the informed (*β* = 0.28, *p* = 0.002) but not the uninformed context (*β* = 0.05, *p* = 0.55). In contrast, parental education predicted math talk diversity in the uninformed (*β* = 0.30, *p* < 0.001) but not the informed context (*β* = 0.10, *p* = 0.27).

Given that our coding system included a greater number of possible numerical (11) than spatial concepts (5), some results—such as the higher frequency of numerical concepts referenced by parents compared to spatial concepts—could reflect an artifact of the coding scheme. To address this potential concern, we conducted a parallel analysis using the proportion of numerical or spatial concepts referenced by parents, calculated as the number of concepts used divided by the total number of available categories within each domain. This analysis produced the same pattern of results: a main effect of context, *F*(1,117) = 120.86, *p* < 0.001, $\eta_{p}^{2}$ = 0.50, with parents referencing a greater proportion of possible math concepts in the informed context (43%) compared to the uninformed context (22%). There was also a main effect of kind of math talk, *F*(1, 117) = 24.73, *p* < 0.001, $\eta_{p}^{2}$ = 0.17, with a higher proportion of possible numeric concepts (35%) being referenced than spatial concepts (26%). Further, there is an interaction between context and kind of math talk, *F*(1, 117) = 162.78, *p* < 0.001, $\eta_{p}^{2}$ = 0.57. Post hoc comparisons indicated that parents used a higher proportion of numeric concepts (out of the total available) in the informed context (53%) compared to the uninformed context (18%, *p* < 0.001, $\eta_{p}^{2}$ = 0.59). In contrast, parents employed a less diverse range of spatial talk in the informed context (22%) compared to the uninformed context (30%, *p* < 0.001, $\eta_{p}^{2}$ = 0.11). These findings support our main predictions that informational priming increases the diversity of parental math talk and that this effect is specific to numerical content.

### 6.3 Parental Regulatory Talk

**FIGURE 2:**
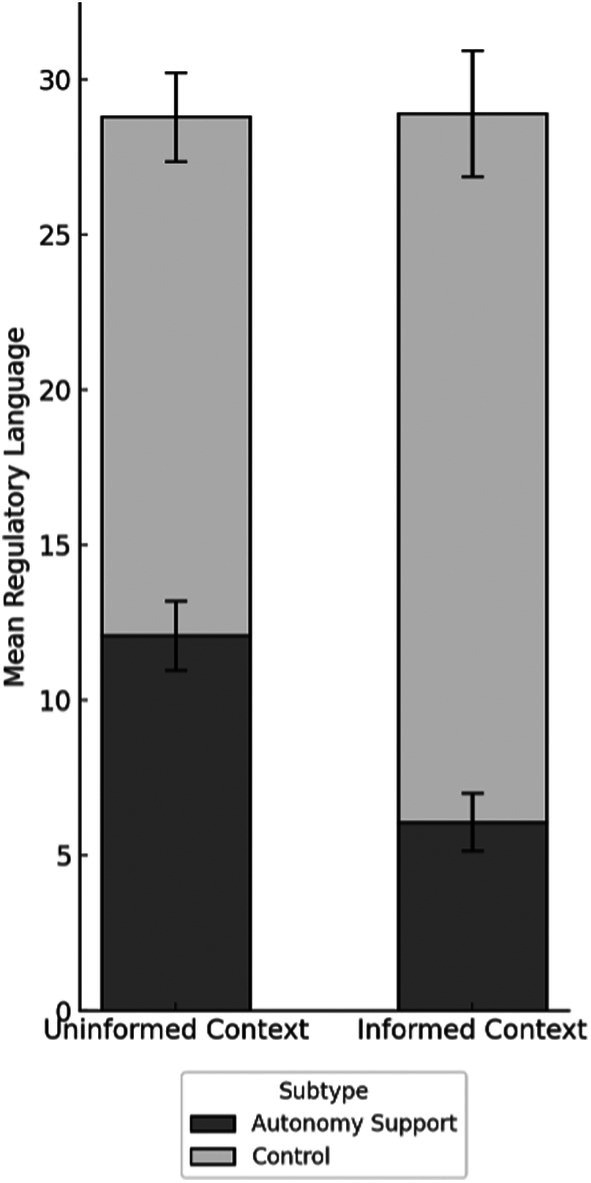
Parental regulatory talk by context and subtype (M, SE).

A 2 (context: uniformed vs. informed) × 2 (kind of regulatory talk: autonomy support vs. control) repeated‐measures ANCOVA was conducted to examine the impact of context on parental autonomy support and control (Hypothesis 3). The results are shown in Figure 2. The analysis revealed no main effect of context, *F*(1, 117) = 0.01, *p* = 0.92, $\eta_{p}^{2}$ = 0.00; parents provided a comparable overall amount of regulatory talk across the uninformed and informed contexts, *M* = 28.80 (SD = 14.56) and *M* = 28.90 (SD = 17.39), respectively. There was a main effect of the kind of regulatory talk, *F*(1, 117) = 78.41, *p* < 0.001, $\eta_{p}^{2}$ = 0.39, with parents using twice as much control as autonomy support across contexts (*M* = 19.78, SD = 1.02 vs. *M* = 9.08, SD = 0.65). Critically, an interaction between context and type of regulatory talk was observed, *F*(1, 117) = 67.73, *p* < 0.001, $\eta_{p}^{2}$ = 0.36. Post hoc comparisons indicated that parents used significantly more control utterances in the informed, compared to the uninformed, context (*p* < 0.001, $\eta_{p}^{2}$ = 0.14). Conversely, they provided significantly less autonomy support in the informed, compared to the uninformed, context (*p* < 0.001, $\eta_{p}^{2}$ = 0.41).

None of the covariates produced main effects on parental regulatory talk. However, the results revealed several interactions. (1) Child age and parental education interacted with context, *F* (1, 117) = 6.76, *p* = 0.01, $\eta_{p}^{2}$ = 0.06; *F* (1, 117) = 3.91, *p* = 0.05, $\eta_{p}^{2}$ = 0.03, respectively. Post hoc analyses (see Table S1E) indicated that in the uninformed context, parental education predicted the overall amount of regulatory talk (*β* = 0.21, *p* = 0.03), while in the informed context, child age was a significant predictor (*β* = −0.24, *p* = 0.01). (2) Both parental education and gender significantly interacted with the type of regulatory talk (*F* (1, 117) = 4.08, *p* = 0.05, $\eta_{p}^{2}$ = 0.03; *F* (1, 117) = 7.30, *p* = 0.008, $\eta_{p}^{2}$ = 0.06, respectively). Post hoc analyses revealed that parental education (*β* = 0.37, *p* = 0.005) but not parental gender (*β* = 0.12, *p* = 0.19) predicted the autonomy support. Conversely, for the amount of control, parental gender (*β* = −2.31, *p* = 0.02) but not parental education (*β* = −0.05, *p* = 0.59) was a significant predictor (see Table S1F).

To control for the loquacity of parents, we conducted a parallel ANCOVA, using the proportion of regulatory talk (out of the total number of utterances) as the outcome variable. The analysis produced the same pattern of results: (a) no overall effect of context, *F*(1, 117) = 0.23, *p* = 0.63, whereby the proportion of regulatory talk was comparable in the uninformed and informed contexts, 9% in both; (b) a main effect of kind of regulatory talk: *F*(1, 117) = 93.72, *p* < 0.001, $\eta_{p}^{2}$ = 0.44, with the proportion of control twice as large as the proportion of autonomy support, 12% versus 6%; and (c) an interaction between context and kind of regulatory talk, *F*(1, 117) = 80.35, *p* < 0.001, $\eta_{p}^{2}$ = 0.40. Follow‐up simple‐effects tests with Bonferroni corrections showed that the proportion of control was greater in the informed, compared to the uninformed context (14% vs. 10%, *p* < 0.001), whereas the proportion of autonomy support was lower in the informed, compared to the uninformed context (4% vs. 7%, *p* < 0.001). These findings support hypothesis 3 that informational priming alters the nature of parental regulatory talk, leading to increased use of control and reduced autonomy support in the informed compared to the uninformed context.

### 6.4 Child (Dis)Engagement

Finally, we examined the impact of context on children's level of disengagement (Hypothesis 4). The ANCOVA revealed a significant main effect of context, *F*(1, 117) = 5.62, *p* = 0.02, $\eta_{p}^{2}$ = 0.04. Aligned with the hypothesis, children exhibited greater disengagement in the informed than in the uninformed context: 11% versus 7%. A significant effect of parent gender was also observed, *F*(1, 117) = 6.68, *p* = 0.01, $\eta_{p}^{2}$ = 0.61, with children being disengaged more frequently when interacting with fathers (16%) than with mothers (8%).

## 7 Discussion

The present study examined the effects of informational priming on the content and the dynamics of parent–child math interactions. Parents engaged with toys in two contexts—uninformed and informed. The informed context involved a “light‐touch” informational priming approach—that is, prior to the play interaction, parents were told about the importance of math for early development in a single sentence. The within‐subject design helped to isolate the effects of information priming, controlling for other individual differences. The results indicated notable inverse effects on parents' math talk (amount and diversity) and interaction dynamics (autonomy support versus control), suggesting trade‐offs between the two constructs. In the sections that follow, we discuss the effects of information priming on each construct and then the implications of the trade‐off observed.

### 7.1 Effects of Informational Priming on Parental Math Talk

The results demonstrated a substantial effect of information priming on parents' math talk. Parents generated an average of four times the number of math utterances and referred to twice as many unique math concepts in the informed than in the uninformed context. Notably, this increase in math talk occurred without a concurrent rise in parental talkativeness, suggesting, as we hypothesized, that the information priming operated via a specific effect on heightened awareness of math opportunities, rather than on overall verbal engagement. This pattern is consistent with prior findings showing that brief instructional prompts can lead to domain‐specific increases in parental input during parent–child math interactions (Zippert et al. 2019).

Further, as expected, information priming had a stronger impact on numerical talk than on spatial talk. Parents generated substantially more utterances related to numerical concepts in the informed compared to the uninformed context, whereas the amount of their spatial talk was comparable across conditions. The observed discrepancy likely reflects parents' tendency to conceptualize math primarily in terms of numbers and arithmetic operations, as reported in prior research (Lu et al. 2025a). Consequently, their efforts to increase math‐related interactions may have been confined to numerical concepts, leading them to overlook opportunities related to spatial reasoning. This imbalance in the effect of information priming, favoring numerical talk, could have long‐term implications for children’ math development by limiting their exposure to a broader range of mathematical concepts (e.g., spatial relations, measurement, and patterns) critical for early math development (Rittle‐Johnson et al. 2019; Verdine et al. 2017). Future research might examine whether this can be offset by explicitly emphasizing the value of spatial reasoning and other math domains in the information prime, such that it produces a more balanced impact on math talk.

Informational priming was also found to interact with parental education. In the uninformed context, parents with more years of education produced a greater diversity of math talk. In contrast, in the informed condition, parental education was not related to differences in math talk. Again, we believe this finding provides support for our hypothesis that informational priming serves to increase parents' awareness of the math opportunities during play interactions. Adults with lower educational attainment may be less attuned to the mathematical aspects of situations, particularly during informal activities where they may be less salient than in formal math situations (Lu et al. 2025b; McMullen et al. 2019). By increasing their awareness of math opportunities, the informational priming may have leveled the playing field across levels of parental education.

This pattern of findings suggests that low‐SES parents are certainly capable of integrating math talk into their interactions with children. Note that in the informed condition, parents were not provided with any specific strategies or guidance on how to engage in math talk; yet they substantially increased both the quantity of math‐related interactions and the diversity of math concepts discussed with children. The fact that they rarely produced math talk in the uninformed context may be partly due to the view of math as a serious academic subject not typically associated with child play. In fact, a recent study showed that parents with less education are not as likely to see playful interactions as relevant to math learning, compared to more educated parents (Lu et al. 2025a). Given that SES‐related variability in parental math talk is theorized to be a key contributor to disparities in early math development (Elliott and Bachman 2018), this study highlights the potential of information‐based interventions as a scalable and effective approach for addressing these disparities.

### 7.2 Effects of Informational Priming on Parent–Child Interaction Dynamics

Informational priming also affected the interaction dynamics—namely, parents' regulatory language and child engagement. While the overall amount of parent regulatory language remained consistent, its composition shifted notably—in the informed context, parents used a greater amount of controlling language and less autonomy support, compared to the uninformed context. Child engagement also differed across conditions, with children exhibiting a greater amount of active disengagement behavior during the informed play interaction than during the uninformed one.

Why did the informational priming have a detrimental effect on parents' autonomy supporting language? One possibility relates to our hypothesized process of the way by which informational priming operates to affect parent behavior. If the informational priming heightened parents' awareness of math opportunities, it may have led them to change their stance toward the activity from an informal play interaction to a more formal academic one. When US parents are asked which pedagogical approach they believe to be most important for their preschool child's home math learning, they are more likely to select a “direct teaching” approach than more informal approaches (Msall et al. 2023). Similarly, when asked about their home math activities, Chinese parents report engaging in formal number‐related activities more frequently than informal ones (Pan et al. 2023). More generally, in Chinese culture, learning is perceived to be largely derived from effort and hard work (Jin 2024), such that parents' own experiences with early math education may have been characterized by repetitive drills or performance‐oriented practices. Increasing parents' awareness of the math aspects of the interaction may have activated these preexisting schemas of early math learning, leading them to transform the playful interaction into a more formal and instructional one. When tasks are framed as academically relevant, parents tend to reduce autonomy support and become more controlling (Grolnick et al. 2002; Huang et al. (2023)). For example, mothers provide preschoolers less autonomy support during a reminiscing task framed as an instructional exercise than as a bonding opportunity. Thus, a change in parents' perception of the purpose of the task may have reduced autonomy‐supportive exchanges.

Another possibility is that the informational priming concurrently triggered or heightened parents' math anxiety, which, in turn, affected their interactional style. When engaging with their children in math activities, parents with high math anxiety have been observed to use more controlling behaviors than those with low math anxiety (Oh et al. 2022; Retanal et al. 2021). Math‐anxious parents also self‐report more negative emotions (e.g., stress) when related to helping their children with math tasks (DiStefano et al. 2020). In the present study, parents, following the informational priming, may have experienced increased anxiety, prompting them to exert more control during the interaction.

A third possibility arises from the decreases in child engagement in the informed, compared to the uninformed, context. As parents began to engage children in more math talk, which potentially involved more frequent corrections of children's responses (Çarkoğlu and Eason 2025), children may have found the activity less playful and, thus, become more disengaged. Children's disengagement behaviors may have then elicited more controlling comments from parents as they attempted to draw children's attention and reengage them. This possibility seems less likely given that the extent of parent math talk was not correlated with child engagement in our study. Rather, it seems more plausible that the primary source of children's increased disengagement was the increase in parents' controlling language, resulting from one of the first two proposed explanations. In fact, it has been established that greater parent control is related to lower levels of child engagement in both the US and China (Chen et al. 2024; Wu et al. 2024).

### 7.3 Implication of the Trade‐Off Between Parent's Math Talk and Regulatory Language

Developmental literature offers accumulating evidence highlighting various trade‐offs that occur in the course of child development. Some trade‐offs emerge as children acquire new skills, with gains in some aspects of their thinking and behavior coming at the expense of other aspects. For example, in the early development of math problem‐solving, there is a well‐documented trade‐off between speed and accuracy when increased efficiency is temporarily associated with decreased accuracy (Siegler and Lemaire 1997). Similarly, increases in selective attention, allowing children to better focus on the target features of the stimuli, may come at the cost of reduced sensitivity to other features that could be relevant to the task (Plebanek and Sloutsky 2017). Another type of trade‐off arises in the context of child‐adult interaction. For example, Bonawitz et al. (2011) found that when adults provided didactic demonstrations emphasizing a single way to use a toy, preschoolers learned that method more quickly but explored less and discovered fewer alternative uses. Note that while some trade‐offs support long‐term success—such as an initial loss in accuracy for improved speed—others may have unintended consequences, such as rapid learning that sacrifices deep, long‐lasting understanding.

The findings of the present study raise concerns about the observed trade‐off in parent–child interactions. Specifically, the increase in parental math talk appears to coincide with more controlling interaction dynamics, a shift that warrants closer scrutiny. Based on prior research, parental autonomy support is considered a stronger and more consistent predictor of children's outcomes, such as academic motivation and long‐term math achievement, than parent math talk (Ryan and Deci 2000). This may help explain why some interventions that successfully increase parental math talk fail to enhance—or sometimes negatively affect—children's math performance. Given these implications, it would be worthwhile for future research on home‐based math interventions to assess changes in multiple facets of parent behavior and to conduct long‐term follow‐ups of the effects on children's math knowledge and interest.

## 8 Limitations and Conclusions

The findings of the current study were consistent with the hypothesized mechanism, whereby informational priming heightens parents' awareness of math opportunities present during interactions and changed the interactional dynamics. Yet, other factors not examined in the study also may have contributed to the results. While the within‐subject design helped control for individual differences, it also introduced a potential confound. The sequence of the two play sessions—uninformed and informed—could not be counterbalanced. Once parents were informed about the importance of math talk, it would not have been feasible to “un‐inform” them. This limitation complicates efforts to isolate the effects of informational priming from a potential familiarity effect whereby parents behaved differently in the informed condition because of increased familiarity with the activity. While steps were taken to mitigate this possibility—including spacing the sessions 2 weeks apart and using different toys—a future investigation could adopt a between subjects' design to better control for it.

In addition, it is possible that the observed increase in parental control was influenced not only by the content of the informational priming, but also by how the message was delivered. Some parents may have perceived the additional instruction as implicit feedback that their initial interaction was lacking, potentially triggering performance‐related anxiety and prompting a more controlling interaction style. Although this interpretation is speculative, it highlights the importance of considering how intervention cues are framed and understood by participants.

Further, it is possible that the informational priming effect observed here might vary based on culture. The present study was conducted with Chinese parents whose child‐rearing beliefs and practices tend to differ from those of Western parents (Huntsinger et al. 1997). Compared to their Western counterparts, Chinese parents place a greater emphasis on math learning (Huntsinger et al. 1997), have higher expectations (Tsui 2005) and are more likely to attribute poor math learning to a lack of effort (Hess et al. 1987). It would be worthwhile for future research to explore the effects of informational priming with more diverse samples and examine potential cultural differences.

Despite these limitations, the present findings highlight the potential of informational priming as a scalable and cost‐effective approach for increasing parental math talk. At the same time, they underscore the need to carefully consider its broader impact on parent–child interactions. While priming successfully increased the quantity and diversity of math talk, it also led to a decline in autonomy support and an increase in controlling behaviors, which in turn was associated with greater child disengagement. Theoretically, the observed trade‐offs point to the need for an integrative framework that accounts for both the content input and interactional dynamic. Practically, interventions that prioritize math input without considering interactional dynamics risk undermining their intended benefits. Moving forward, designing interventions that not only enhance math talk but also support autonomy and engagement will be critical to fostering both cognitive and motivational aspects of children's math development.

## Supporting information


**Data S1:** cdev70031‐sup‐0001‐supinfo.docx.

## Data Availability

The data necessary to reproduce the analyses presented here are available upon request. The analytic code necessary to reproduce the analyses presented in this paper is available upon request. The materials and analytic code necessary to attempt to replicate the findings presented here are available upon request. The analyses presented here were not preregistered.

## References

[cdev70031-bib-0001] Aunola, K. , J. Viljaranta , E. Lehtinen , and J. E. Nurmi . 2013. “The Role of Maternal Support of Competence, Autonomy and Relatedness in Children's Interests and Mastery Orientation.” Learning and Individual Differences 25: 171–177. 10.1016/j.lindif.2013.02.002.

[cdev70031-bib-0002] Bachman, H. J. , J. L. Degol , L. Elliott , L. Scharphorn , N. E. El Nokali , and K. M. Palmer . 2018. “Preschool Math Exposure in Private Center‐Based Care and Low‐SES Children's Math Development.” Early Education and Development 29, no. 3: 417–434. 10.1080/10409289.2017.1406245.

[cdev70031-bib-0003] Berkowitz, T. , D. J. Gibson , and S. C. Levine . 2021. “Parent Math Anxiety Predicts Early Number Talk.” Journal of Cognition and Development 22, no. 4: 523–536. 10.1080/15248372.2021.1926252. 34335106 PMC8318342

[cdev70031-bib-0004] Berkowitz, T. , M. W. Schaeffer , E. A. Maloney , et al. 2015. “Math at Home Adds up to Achievement in School.” Science 350, no. 6257: 196–198. 10.1126/science.aac7427. 26450209

[cdev70031-bib-0005] Bonawitz, E. , P. Shafto , H. Gweon , N. D. Goodman , E. Spelke , and L. Schulz . 2011. “The Double‐Edged Sword of Pedagogy: Instruction Limits Spontaneous Exploration and Discovery.” Cognition 120, no. 3: 322–330. 10.1016/j.cognition.2010.10.001. 21216395 PMC3369499

[cdev70031-bib-0006] Braham, E. J. , M. E. Libertus , and K. McCrink . 2018. “Children's Spontaneous Focus on Number Before and After Guided Parent–Child Interactions in a Children's Museum.” Developmental Psychology 54, no. 8: 1492–1498. 10.1037/dev0000534. 30047774 PMC6132254

[cdev70031-bib-0007] Çarkoğlu, C. , and S. H. Eason . 2025. “How Does Activity Context Relate to Parents' Responses to Preschoolers' Errors and Correct Math Statements?” Journal of Experimental Child Psychology 253: 106191. 10.1016/j.jecp.2024.106191. 39892332

[cdev70031-bib-0008] Casey, B. M. , C. M. Lombardi , D. Thomson , et al. 2018. “Maternal Support of Children's Early Numerical Concept Learning Predicts Preschool and First‐Grade Math Achievement.” Child Development 89, no. 1: 156–173. 10.1111/cdev.12676. 27861760

[cdev70031-bib-0009] Chen, X. , N. L. McElwain , E. M. Pomerantz , and M. Wang . 2024. “Maternal Autonomy Support and Intrusive Control in the United States and China: Moment‐To‐Moment Associations With Preschoolers' Agency and Defeat.” Developmental Psychology 60, no. 6: 1016–1027. 10.1037/dev0001723. 38421784

[cdev70031-bib-0010] Cheung, S. K. , and C. McBride . 2017. “Effectiveness of Parent–Child Number Board Game Playing in Promoting Chinese Kindergarteners' Numeracy Skills and Mathematics Interest.” Early Education and Development 28, no. 5: 572–589. 10.1080/10409289.2016.1258932.

[cdev70031-bib-0011] Daucourt, M. C. , A. R. Napoli , J. M. Quinn , S. G. Wood , and S. A. Hart . 2021. “The Home Math Environment and Math Achievement: A Meta‐Analysis.” Psychological Bulletin 147, no. 6: 565–596. 10.1037/bul0000330. 34843299 PMC8634776

[cdev70031-bib-0012] Dearing, E. , B. Casey , P. E. Davis‐Kean , et al. 2022. “Socioeconomic Variations in the Frequency of Parent Number Talk: A Meta‐Analysis.” Education in Science 12, no. 5: 312. 10.3390/educsci12050312. PMC10811961 38282965

[cdev70031-bib-0013] DeFlorio, L. , and A. Beliakoff . 2015. “Socioeconomic Status and Preschoolers' Mathematical Knowledge: The Contribution of Home Activities and Parent Beliefs.” Early Education and Development 26, no. 3: 319–341. 10.1080/10409289.2015.968239.

[cdev70031-bib-0014] DiStefano, M. , B. O'Brien , A. Storozuk , G. Ramirez , and E. A. Maloney . 2020. “Exploring Math Anxious Parents' Emotional Experience Surrounding Math Homework‐Help.” International Journal of Educational Research 99: 101526. 10.1016/j.ijer.2019.101526.

[cdev70031-bib-0015] Distefano, R. , and A. S. Meuwissen . 2022. “Parenting in Context: A Systematic Review of the Correlates of Autonomy Support.” Journal of Family Theory & Review 14, no. 4: 571–592. 10.1111/jftr.12465.

[cdev70031-bib-0016] Doss, C. , H. Fricke , S. Loeb , and J. B. Doromal . 2022. “Engaging Girls in Math: The Unequal Effects of Text Messaging to Help Parents Support Early Math Development.” Economics of Education Review 88: 102262. 10.1016/j.econedurev.2022.102262.

[cdev70031-bib-0017] Douglas, A. A. , C. Msall , F. Logan , and B. Rittle‐Johnson . 2024. “The Impact of Brief Information‐Based Interventions on the Home Math Environment.” Journal of Applied Developmental Psychology 94: 101682. 10.1016/j.appdev.2024.101682.

[cdev70031-bib-0018] Dulay, K. M. , S. K. Cheung , P. Reyes , and C. McBride . 2019. “Effects of Parent Coaching on Filipino Children's Numeracy, Language, and Literacy Skills.” Journal of Educational Psychology 111, no. 4: 641–662. 10.1037/edu0000315.

[cdev70031-bib-0019] Duncan, G. J. , C. J. Dowsett , A. Claessens , et al. 2007. “School Readiness and Later Achievement.” Developmental Psychology 43, no. 6: 1428–1446. 10.1037/0012-1649.43.6.1428. 18020822

[cdev70031-bib-0020] Duncan, G. J. , and K. Magnuson . 2011. “The Nature and Impact of Early Achievement Skills, Attention Skills, and Behavior Problems.” In Whither Opportunity, edited by G. J. Duncan and R. J. Murnane , 47–70. Sage Publications. 10.1037/0012-1649.43.6.1428.

[cdev70031-bib-0021] Eason, S. H. , and G. B. Ramani . 2020. “Parent–Child Math Talk About Fractions During Formal Learning and Guided Play Activities.” Child Development 91, no. 2: 546–562. 10.1111/cdev.13199. 30566248

[cdev70031-bib-0022] Elliott, L. , and H. J. Bachman . 2018. “SES Disparities in Early Math Abilities: The Contributions of Parents' Math Cognitions, Practices to Support Math, and Math Talk.” Developmental Review 49: 1–15. 10.1016/j.dr.2018.08.001.

[cdev70031-bib-0078] Faul, F. , E. Erdfelder , A. G. Lang , and A. Buchner . 2007. “G*Power 3: A flexible statistical power analysis program for the social, behavioral, and biomedical sciences.” Behavior Research Methods 39, no. 2: 175–191. 10.3758/BF03193146. 17695343

[cdev70031-bib-0023] Ferrara, K. , K. Hirsh‐Pasek , N. S. Newcombe , R. M. Golinkoff , and W. S. Lam . 2011. “Block Talk: Spatial Language During Block Play.” Mind, Brain, and Education 5, no. 3: 143–151. 10.1111/j.1751-228X.2011.01122.x.

[cdev70031-bib-0024] Gao, Y. 2024. “Overcoming Parenting Barriers in Under‐Resourced Communities With Evidence From a Field Experiment in Rural China.” 10.2139/ssrn.4969618.

[cdev70031-bib-0025] Gibson, D. J. , E. A. Gunderson , and S. C. Levine . 2020. “Causal Effects of Parent Number Talk on Preschoolers' Number Knowledge.” Child Development 91, no. 6: e1162–e1177. 10.1111/cdev.13423. 33164211 PMC10683715

[cdev70031-bib-0026] Grolnick, W. S. , S. T. Gurland , W. DeCourcey , and K. Jacob . 2002. “Antecedents and Consequences of Mothers' Autonomy Support: An Experimental Investigation.” Developmental Psychology 38, no. 1: 143–155. 10.1037//0012-1649.38.1.143. 11806696

[cdev70031-bib-0027] Hagger, M. S. , S. Sultan , S. J. Hardcastle , and N. L. Chatzisarantis . 2015. “Perceived Autonomy Support and Autonomous Motivation Toward Mathematics Activities in Educational and Out‐Of‐School Contexts Is Related to Mathematics Homework Behavior and Attainment.” Contemporary Educational Psychology 41: 111–123. 10.1016/j.cedpsych.2014.12.002.

[cdev70031-bib-0028] Hanner, E. , E. J. Braham , L. Elliott , and M. E. Libertus . 2019. “Promoting Math Talk in Adult–Child Interactions Through Grocery Store Signs.” Mind, Brain, and Education 13, no. 2: 110–118. 10.1111/mbe.12195.

[cdev70031-bib-0029] Hess, R. D. , C. Chang , and T. M. McDevitt . 1987. “Cultural Variations in Family Beliefs About Children's Performance in Mathematics: Comparisons Among People's Republic of China, Chinese‐American, and Caucasian‐American Families.” Journal of Educational Psychology 79, no. 2: 179–188. 10.1037//0022-0663.79.2.179.

[cdev70031-bib-0030] Huang, Q. , J. Sun , E. Y. H. Lau , and Y. L. Zhou . 2023. “Parental Scaffolding and Children's Math Ability: The Type of Activities Matters.” British Journal of Developmental Psychology 41, no. 3: 246–258. 10.1111/bjdp.12444. 36859815

[cdev70031-bib-0031] Huntsinger, C. S. , P. E. Jose , F. R. Liaw , and W. D. Ching . 1997. “Cultural Differences in Early Mathematics Learning: A Comparison of Euro‐American, Chinese‐American, and Taiwan‐Chinese Families.” International Journal of Behavioral Development 21, no. 2: 371–388.

[cdev70031-bib-0033] Huttenlocher, J. , H. Waterfall , M. Vasilyeva , J. Vevea , and L. V. Hedges . 2010. “Sources of Variability in Children's Language Growth.” Cognitive Psychology 61, no. 4: 343–365. 10.1016/j.cogpsych.2010.08.002. 20832781 PMC2981670

[cdev70031-bib-0034] Jin, X. 2024. “The Role of Effort in Understanding Academic Achievements: Empirical Evidence From China.” European Journal of Psychology of Education 39, no. 1: 389–409. 10.1007/s10212-023-00694-5.

[cdev70031-bib-0035] Klibanoff, R. S. , S. C. Levine , J. Huttenlocher , M. Vasilyeva , and L. V. Hedges . 2006. “Preschool Children's Mathematical Knowledge: The Effect of Teacher “Math Talk”.” Developmental Psychology 42, no. 1: 59–69. 10.1037/0012-1649.42.1.59. 16420118

[cdev70031-bib-0036] Koo, T. K. , and M. Y. Li . 2016. “A Guideline of Selecting and Reporting Intraclass Correlation Coefficients for Reliability Research.” Journal of Chiropractic Medicine 15, no. 2: 155–163. 10.1016/j.jcm.2016.02.012. 27330520 PMC4913118

[cdev70031-bib-0037] Kulkofsky, S. 2011. “Characteristics of Functional Joint Reminiscence in Early Childhood.” Memory 19, no. 1: 45–55. 10.1080/09658211.2010.535542. 21154014

[cdev70031-bib-0038] Larson, K. , S. A. Russ , B. B. Nelson , L. M. Olson , and N. Halfon . 2015. “Cognitive Ability at Kindergarten Entry and Socioeconomic Status.” Pediatrics 135, no. 2: e440–e448. 10.1542/peds.2014-0434. 25601983

[cdev70031-bib-0039] Laurin, J. C. , and M. Joussemet . 2017. “Parental Autonomy‐Supportive Practices and Toddlers' Rule Internalization: A Prospective Observational Study.” Motivation and Emotion 41: 562–575. 10.1007/s11031-017-9627-5.

[cdev70031-bib-0040] Levine, S. C. , K. R. Ratliff , J. Huttenlocher , and J. Cannon . 2012. “Early Puzzle Play: A Predictor of Preschoolers' Spatial Transformation Skill.” Developmental Psychology 48, no. 2: 530–542. 10.1037/a0025913. 22040312 PMC3289766

[cdev70031-bib-0041] Leyva, D. , M. E. Libertus , and R. McGregor . 2023. “Math Intervention Targeting Family Routines Increases Parental Math Talk and Math Activities.” Journal of Applied Developmental Psychology 89: 101595. 10.1016/j.appdev.2023.101595.

[cdev70031-bib-0042] Lombardi, C. M. , and E. Dearing . 2021. “Maternal Support of Children's Math Learning in Associations Between Family Income and Math School Readiness.” Child Development 92, no. 1: e39–e55. 10.1111/cdev.13436. 32797635

[cdev70031-bib-0043] Loulis, S. , and L. Kuczynski . 1997. “Beyond One Hand Clapping: Seeing Bidirectionality in Parent‐Child Relations.” Journal of Social and Personal Relationships 14, no. 4: 441–461. 10.1177/0265407597144002.

[cdev70031-bib-0044] Lu, L. , M. Vasilyeva , and E. V. Laski . 2023. “Minor Changes, Big Differences? Effects of Manipulating Play Materials on Parental Math Talk.” Developmental Psychology 59, no. 7: 1283–1299. 10.1037/dev0001550. 37199928

[cdev70031-bib-0045] Lu, L. , M. Vasilyeva , and E. V. Laski . 2025a. “Home Math Environment as a Mediator of Socioeconomic Differences in Early Math Skills: A Study of Chinese Families From Disparate Backgrounds.” Developmental Psychology 61, no. 3: 417–431. 10.1037/dev0001918. 39847019

[cdev70031-bib-0046] Lu, L. , M. Vasilyeva , and E. V. Laski . 2025b. “Spontaneous Focus on Numerosity in Parents of Preschoolers: Is It Related to Actual Math Input Parents Provide?” Journal of Experimental Child Psychology 250: 106121. 10.1016/j.jecp.2024.106121. 39546847

[cdev70031-bib-0047] Mayer, S. E. , A. Kalil , W. Delgado , H. Liu , D. Rury , and R. Shah . 2023. “Boosting Parent‐Child Math Engagement and Preschool Children's Math Skills: Evidence From an RCT With Low‐Income Families.” Economics of Education Review 95: 102436. 10.1016/j.econedurev.2023.102436.

[cdev70031-bib-0048] McMullen, J. , J. Y. C. Chan , M. M. Mazzocco , and M. M. Hannula‐Sormunen . 2019. “Spontaneous Mathematical Focusing Tendencies in Mathematical Development and Education.” In Constructing Number: Merging Perspectives From Psychology and Mathematics Education, 69–86. Springer International Publishing. 10.1007/978-3-030-00491-0_4.

[cdev70031-bib-0049] Msall, C. , A. A. Douglas , and B. Rittle‐Johnson . 2023. “Parents' Approaches to Numeracy Support: What Parents Do Is Rarely What They Think Is Most Important.” Frontiers in Education 8: 1114803. 10.3389/feduc.2023.1114803.

[cdev70031-bib-0050] Oh, D. D. , M. M. Barger , and E. M. Pomerantz . 2022. “Parents' Math Anxiety and Their Controlling and Autonomy‐Supportive Involvement in Children's Math Learning: Implications for Children's Math Achievement.” Developmental Psychology 58, no. 11: 2158–2170. 10.1037/dev0001422. 35951395

[cdev70031-bib-0051] Pan, Y. , M. Gauvain , Z. Liu , and L. Cheng . 2006. “American and Chinese Parental Involvement in Young Children's Mathematics Learning.” Cognitive Development 21, no. 1: 17–35. 10.1016/j.cogdev.2005.08.001.

[cdev70031-bib-0052] Pan, Y. , B. Y. Hu , J. Hunt , Z. Wu , Y. Chen , and M. He . 2023. “Chinese Preschool Children's Home Numeracy Experiences and Their Mathematical Abilities.” Journal of Early Childhood Research 21, no. 1: 31–45. 10.1177/1476718X221125583.

[cdev70031-bib-0053] Park, H. , and A. S. Lau . 2016. “Socioeconomic Status and Parenting Priorities: Child Independence and Obedience Around the World.” Journal of Marriage and Family 78, no. 1: 43–59. 10.1111/jomf.12247.

[cdev70031-bib-0054] Plebanek, D. J. , and V. M. Sloutsky . 2017. “Costs of Selective Attention: When Children Notice What Adults Miss.” Psychological Science 28, no. 6: 723–732. 10.1177/0956797617711664. 28388275 PMC5461181

[cdev70031-bib-0055] Purpura, D. J. , S. A. Schmitt , A. R. Napoli , et al. 2021. “Engaging Caregivers and Children in Picture Books: A Family‐Implemented Mathematical Language Intervention.” Journal of Educational Psychology 113, no. 7: 1338–1353. 10.1037/edu0000662.

[cdev70031-bib-0056] Ramani, G. B. , M. L. Rowe , S. H. Eason , and K. A. Leech . 2015. “Math Talk During Informal Learning Activities in Head Start Families.” Cognitive Development 35: 15–33. 10.1016/j.cogdev.2014.11.002.

[cdev70031-bib-0057] Retanal, F. , N. B. Johnston , S. M. Di Lonardo Burr , A. Storozuk , M. DiStefano , and E. A. Maloney . 2021. “Controlling‐Supportive Homework Help Partially Explains the Relation Between Parents' Math Anxiety and Children's Math Achievement.” Education in Science 11, no. 10: 620. 10.3390/educsci11100620.

[cdev70031-bib-0058] Ribeiro, L. A. , B. Casey , E. Dearing , K. B. Nordahl , C. Aguiar , and H. Zachrisson . 2020. “Early Maternal Spatial Support for Toddlers and Math Skills in Second Grade.” Journal of Cognition and Development 21, no. 2: 282–311. 10.1080/15248372.2020.1717494.

[cdev70031-bib-0059] Rispoli, K. M. , N. A. Koziol , K. E. McGoey , and J. B. Schreiber . 2019. “Parenting, Childcare, and Children's Pre‐Kindergarten Skills: Exploring Moderation by Race and Ethnicity.” Early Child Development and Care 189, no. 6: 946–964. 10.1080/03004430.2017.1359580.

[cdev70031-bib-0060] Rittle‐Johnson, B. , E. L. Zippert , and K. L. Boice . 2019. “The Roles of Patterning and Spatial Skills in Early Mathematics Development.” Early Childhood Research Quarterly 46: 166–178. 10.1016/j.ecresq.2018.03.006.

[cdev70031-bib-0061] Ryan, R. M. , and E. L. Deci . 2000. “Self‐Determination Theory and the Facilitation of Intrinsic Motivation, Social Development, and Well‐Being.” American Psychologist 55, no. 1: 68–78. 10.1037/0003-066X.55.1.68. 11392867

[cdev70031-bib-0062] Scalise, N. R. , K. Pak , M. Arrington , and G. B. Ramani . 2025. “Early Mathematics Instruction and Teachers' Self‐Efficacy Beliefs: A Mixed‐Methods Investigation.” Early Childhood Education Journal 53, no. 4: 1119–1132.

[cdev70031-bib-0063] Siegler, R. S. , and P. Lemaire . 1997. “Older and Younger Adults' Strategy Choices in Multiplication: Testing Predictions of ASCM Using the Choice/No‐Choice Method.” Journal of Experimental Psychology: General 126, no. 1: 71–92. 10.1037/0096-3445.126.1.71. 9090145

[cdev70031-bib-0064] Silinskas, G. , and E. Kikas . 2019. “Parental Involvement in Math Homework: Links to Children's Performance and Motivation.” Scandinavian Journal of Educational Research 63, no. 1: 17–37. 10.1080/00313831.2017.1324901.

[cdev70031-bib-0065] Son, S. H. C. , and J. H. Hur . 2020. “Parental Math Talk During Home Cooking and Math Skills in Head Start Children: The Role of Task Management Talk.” Journal of Research in Childhood Education 34, no. 3: 406–426. 10.1080/02568543.2019.1704318.

[cdev70031-bib-0066] Su, Y. , H. S. Doerr , W. Johnson , J. Shi , and F. M. Spinath . 2015. “The Role of Parental Control in Predicting School Achievement Independent of Intelligence.” Learning and Individual Differences 37: 203–209. 10.1016/j.lindif.2014.11.023.

[cdev70031-bib-0067] Susperreguy, M. I. , and P. E. Davis‐Kean . 2016. “Maternal Math Talk in the Home and Math Skills in Preschool Children.” Early Education and Development 27, no. 6: 841–857. 10.1080/10409289.2016.1148480.

[cdev70031-bib-0079] Tian, J. , G. Bennett‐Pierre , N. Tavassolie , et al. 2025. “A Month‐Long Parent‐Led Spatial Intervention Failed to Improve Children's Spatial Skills.” Mind, Brain, and Education. 10.1111/mbe.70013.

[cdev70031-bib-0069] Tsui, M. 2005. “Family Income, Home Environment, Parenting, and Mathematics Achievement of Children in China and the United States.” Education and Urban Society 37, no. 3: 336–355. 10.1177/0013124504274188.

[cdev70031-bib-0070] Vandermaas‐Peeler, M. , E. Boomgarden , L. Finn , and C. Pittard . 2012. “Parental Support of Numeracy During a Cooking Activity With Four‐Year‐Olds.” International Journal of Early Years Education 20, no. 1: 78–93. 10.1080/09669760.2012.663237.

[cdev70031-bib-0071] Vandermaas‐Peeler, M. , J. Nelson , C. Bumpass , and B. Sassine . 2009. “Numeracy‐Related Exchanges in Joint Storybook Reading and Play.” International Journal of Early Years Education 17, no. 1: 67–84. 10.1080/09669760802699910.

[cdev70031-bib-0072] Verdine, B. N. , R. M. Golinkoff , K. Hirsh‐Pasek , and N. S. Newcombe . 2017. “I. Spatial Skills, Their Development, and Their Links to Mathematics.” Monographs of the Society for Research in Child Development 82, no. 1: 7–30. 10.1111/mono.12280. 28181248

[cdev70031-bib-0073] Wang, M. , M. Vasilyeva , and E. V. Laski . 2024. “Words Matter: Effect of Manipulating Storybook Texts on Parent and Child Math Talk.” Early Childhood Research Quarterly 69: 65–77. 10.1016/j.ecresq.2024.07.002.

[cdev70031-bib-0074] Wood, B. K. , R. L. Hojnoski , S. D. Laracy , and C. L. Olson . 2016. “Comparison of Observational Methods and Their Relation to Ratings of Engagement in Young Children.” Topics in Early Childhood Special Education 35, no. 4: 211–222. 10.1177/0271121414565911.

[cdev70031-bib-0075] Wu, J. , D. Oh , D. C. Hyde , and E. M. Pomerantz . 2024. “Cognitive and Motivational Numeracy Parenting Practices: Implications for Children's Numeracy Engagement During Early Elementary School.” Developmental Psychology 60, no. 4: 680–692. 10.1037/dev0001706. 38358666

[cdev70031-bib-0076] Zippert, E. L. , E. N. Daubert , N. R. Scalise , G. D. Noreen , and G. B. Ramani . 2019. “‘Tap Space Number Three’: Promoting Math Talk During Parent‐Child Tablet Play.” Developmental Psychology 55, no. 8: 1605–1614. 10.1037/dev0000769. 31192643

[cdev70031-bib-0077] Zippert, E. L. , and B. Rittle‐Johnson . 2020. “The Home Math Environment: More Than Numeracy.” Early Childhood Research Quarterly 50: 4–15.

