# Supplementary material for: Navigating Trade‐Offs in Early Math: How Informational Priming Influences Parent–Child Interaction
Source: Child Dev. 2025 Aug 21;96(6):2162–75. doi: 10.1111/cdev.70031 (PMC12598456; doi:10.1111/cdev.70031)
Supplement: Supplementary file 1 — Data S1: cdev70031‐sup‐0001‐supinfo.docx. [file CDEV-96-2162-s001.docx]

**Figure S1A.**

*Play Materials and Parental Instruction in the Two Contexts*

|  | **Uninformed Context** | **Informed Context** |
| --- | --- | --- |
| **Play Materials** | 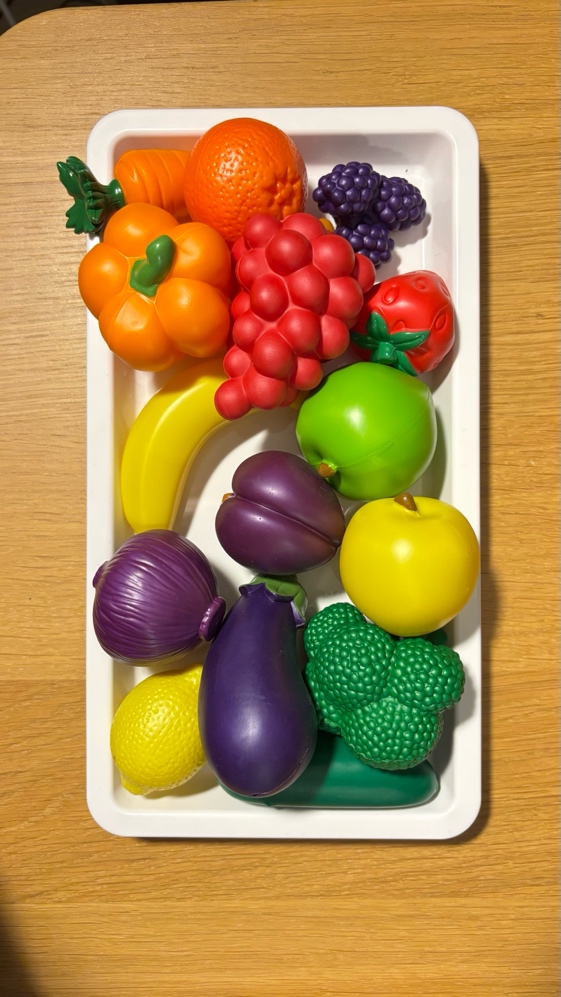 | 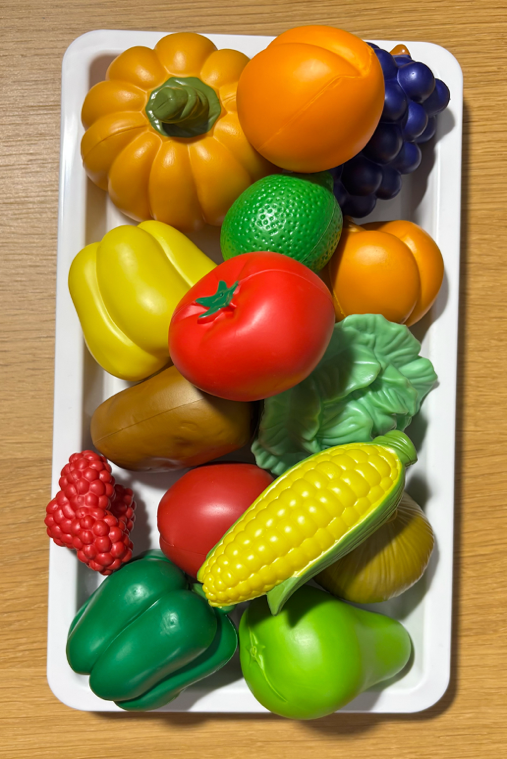 |
| **Parental Instruction (prior to the session)** | “Here are some toys — you and your child could use them to pretend that you are having a party together!  You may discuss what you would like to put on the plate.  Play as you typically would.” | “Talking about math during play is really helpful for children’s early math development.  Here are some toys — you and your child could use them to pretend that you are having a party together! You may discuss what you would like to put on the plate.  Play as you typically would.” |

**Table S1A.**

*Types of Math Talk (Descriptions, Examples, and Descriptive Statistics across Two Contexts)*

| **Type of math talk** | **Description and example** | ***M* (*SD*)** | | **ICC** |
| --- | --- | --- | --- | --- |
|  |  | **Uninformed** | **Informed** |  |
| *Numeric Talk* |  |  |  |  |
| Counting | Comment providing or requesting different ways of counting  (e.g., “Can you count how many fruits are there?”) | .62  (1.41) | 6.09  (6.24) | .95 |
| Number-naming | Comment providing or requesting numbers without observed quantities  (e.g., “let’s set a price for each type of the fruits!”) | .86  (2.37) | 1.89  (4.98) | .98 |
| Cardinality | Comment about the number of observed objects  (e.g., “we have five fruits!”) | 2.53  (3.70) | 18.41  (12.70) | .87 |
| Vague quantifiers | Comment about “a lot” or “a few” without referencing the cardinal value of the set  (e.g., “You have a lot of fruits!”) | .80  (1.38) | .80  (1.70) | .90 |
| Number comparison | Comment about the comparison between specific numbers or quantities  (e.g., “You have five fruits, I have three. Yours are more than mine. ”) | .36  (1.29) | 1.36  (2.99) | .82 |
| Addition | Comment modeling or requesting addition operations  (e.g., “You have 5 fruits, I give you 3 more, how many do we have in total?”) | .21  (.90) | 4.24  (5.97) | .88 |
| Subtraction | Comment modeling or requesting subtraction operations  (e.g., “You have 5 fruits, I take away 3, how many do you have left?”) | .09  (.62) | 3.21  (4.86) | .88 |
| Decomposition | Comment breaking down a complex arithmetic problem into smaller components  (e.g., “How to use get-to-ten strategy to add 7 and 6 fruits together?”) | .04  (.45) | .27  (1.39) | 1.00 |
| Multiplication | Comment modeling or requesting multiplication operations  (e.g., “Each of the five guests wants two fruits, how many should we get in total?”) | .00  (.00) | .07  (.37) | 1.00 |
| Division | Comment modeling or requesting multiplication operations  (e.g., “How should we divide the six fruits for our three guests?”) | .02  (.20) | .48  (1.93) | .99 |
| Other numeric  concepts | Other numeric-related comments include but not limited to fractions or proportions, negative numbers (e.g., “You only have 3 fruits, I want 4. You own me 1, negative 1.”) | .20  (.74) | 2.96  (4.80) | .89 |
| *Spatial Talk* |  |  |  |  |
| Shape | Comment discussing the shape of objects (e.g., “what shape is this fruit?”) | .31 (1.05) | .27 (1.11) | .99 |
| Categorization | Comment categorizing objects (e.g., “let’s group them into two sets!”) | 5.43 (5.99) | 5.48(6.07) | .98 |
| Size | Comment discussing the size of objects (e.g., “the pear is bigger than this apple.”) | .73 (1.59) | .80 (4.46) | .95 |
| Pattern | Comment discussing patterns of arrangement (e.g., “let’s set the toys up in a pattern alternating one large with one small.”) | .06 (.35) | .10 (.55) | .87 |
| Other spatial  concepts | Other spatial-related comments such as the orientation of objects (e.g., let’s lay out the grape horizontally and eggplant vertically.”) | .43 (1.17) | .40 (2.07) | .97 |

**Table S1B**

*Types of Regulatory Talk and Child Disengagement (Descriptions, Examples, and Descriptive Statistics across Two Contexts)*

| **Measures** | **Description and example** | ***M* (*SD*)** | | **ICC** |
| --- | --- | --- | --- | --- |
|  |  | ***Uninformed*** | ***Informed*** |  |
| *Regulatory Talk* | | | | |
| Autonomy Support | Comment providing child agency by giving the child the opportunity to lead or take control in the activity or make decisions.  (e.g., “What do you want to do next?”) | 12.08  (8.68) | 6.08  (7.17) | .93 |
| Control | Parent undermines child’s agency by giving a command to change the kids’ behavior without justification. Or parent makes a clear attempt to control children’s behavior or ignores children’s opinions.  (e.g., “No, you cannot touch the apple.”) | 16.72  (11.16) | 22.83  (15.81) | .94 |
|  |  |  |  |  |
| *Child Disengagement* | | | | |
| % of Time | Verbal or behavioral indications that the child is disengaged from the ongoing activity.  (e.g., looking at or pointing to unrelated objects in the room, walking away from the activity, introducing unrelated conversation topics such as “Can we have pizza tonight?”, or not responding to the parent's requests or questions). | 7.34  (12.96) | 10.98  (18.72) | .87 |

**Table S1C**

*Regression Analyses of Parent’s Total Utterances in Two Contexts*

| **Dependent Variable** | **Uninformed Context** | | **Informed Context** | |
| --- | --- | --- | --- | --- |
|  | $\beta$ | *p* | $\beta$ | *p* |
| Child Gender | -.00 | .96 | .16 | .88 |
| Child Age | -.12 | .17 | -.07 | .46 |
| Parent Gender | -.16 | .06 | -.10 | .25 |
| Parent Education | .42 | < .001 | .20 | .03 |
| Overall Model Fit | Adjusted $R^{2}$ = .19, *p* < .001 | | Adjusted $R^{2}$ = .03, *p* = .14 | |

***Note.*** Parent and child gender were coded 1 = male, 0 = female.

**Table S1D**

*Regression Analyses of Parent’s Math Talk (Diversity) in Two Contexts*

| **Dependent Variable** | **Uninformed Context** | | **Informed Context** | |
| --- | --- | --- | --- | --- |
|  | $\beta$ | *p* | $\beta$ | *p* |
| Child Gender | .07 | .41 | -.09 | .31 |
| Child Age | .05 | .55 | .28 | .002 |
| Parent Gender | -.23 | .008 | -.08 | .36 |
| Parent Education | .30 | < .001 | .10 | .27 |
| Overall Model Fit | Adjusted $R^{2}$ = .10, *p* = .002 | | Adjusted $R^{2}$ = .07, *p* = .02 | |

***Note.*** Parent and child gender were coded 1 = male, 0 = female.

**Table S1E**

*Regression Analyses of Parent’s Overall Regulatory Talk in Two Contexts*

| **Dependent Variable** | **Uninformed Context** | | **Informed Context** | |
| --- | --- | --- | --- | --- |
|  | $\beta$ | *P* | $\beta$ | *p* |
| Child Gender | -.02 | .84 | -.04 | .62 |
| Child Age | -.01 | .96 | -.24 | .01 |
| Parent Gender | -.12 | .20 | -.07 | .42 |
| Parent Education | .21 | .03 | -.02 | .86 |
| Overall Model Fit | Adjusted $R^{2}$ = .02, *p* = .18 | | Adjusted $R^{2}$ = .03, *p* = .12 | |

***Note*.** Parent and child gender were coded 1 = male, 0 = female.

**Table F.**

*Regression Analyses of Two Types of Regulatory Talk across Two Contexts*

| **Dependent Variable** | **Control** | | **Autonomy Support** | |
| --- | --- | --- | --- | --- |
|  | $\beta$ | *p* | $\beta$ | *p* |
| Child Gender | -.07 | .42 | .03 | .71 |
| Child Age | -.14 | .13 | -.07 | .45 |
| Parent Gender | -2.31 | .02 | .12 | .19 |
| Parent Education | -.05 | .59 | .37 | .005 |
| Overall Model Fit | Adjusted $R^{2}$ = .04, *p* = .09 | | Adjusted $R^{2}$ = .07, *p* = .02 | |

***Note*.** Parent and child gender were coded 1 = male, 0 = female.
